# Health expenditure, longevity, and child mortality: dynamic panel data approach with global data

**DOI:** 10.1007/s10754-019-09272-z

**Published:** 2019-09-06

**Authors:** Devdatta Ray, Mikael Linden

**Affiliations:** Department of Health and Social Management (Health Economics), University of Eastern Finland (UEF Kuopio Campus), P.O. Box 162, 70211 Kuopio, Finland

**Keywords:** Health expenditures, Low and high incomes, Life expectancy, Dynamic panel methods, I15, I18, H51, C30

## Abstract

In this study, effects of public and private health expenditures on life expectancy at birth and infant mortality are analysed on a global scale with 195 countries in the years 1995–2014. The global data set is divided into country categories according to growth in life expectancy, decrease in infant mortality rate, and level of gross national income per capita. Some new dynamic panel model estimators, argued to be more efficient with high persistence series and predetermination compared to popular but complex *GMM* estimators, show that public health expenditures are generally more health-promoting than private expenditures. However, the health effects are not as great as primary education effects. Although the new estimators provide some new and valuable information on health expenditure effects on life expectancy and infant mortality on a global scale, they do not show desired robustness.

## Introduction

Globally, health spending is highly unequal. It is even more unequally distributed than the national income of countries (Deaton 2013). Countries that spend little on health also have poorer health conditions. Although OECD countries have less than 20% of the world’s population, they accounted for over 80% of world spending on health at the turn of the new century, whereas the poorest three quarters of the world’s population accounted for only 7% of the world’s health expenditures (*HE*). Looking across regions, Africa contains about 10% of the world’s population, yet it accounted for 3% of the world’s health spending. Asia and the Pacific (including China), with almost 30% of the world’s population, accounted for only 4% of the world’s health spending (OECD 2014; Poullier et al. 2002; WHO 2015).

Although health spending should affect health conditions, it is important to note that the efficiency of transforming spending into better health outcomes varies significantly. Even at low levels of *HE* per capita spending, some countries achieve better health than others. This means that there may be an opportunity for public policy to also make a difference. On the contrary, among high-spending countries, sometimes additional spending bears little relationship to improvements in life expectancy. This is one reason behind the concern in wealthier countries for cost containment.

During most of the second half and especially the last decades of the twentieth century, public *HE* grew at a faster rate than the national income in developed countries. Empirical studies show that demographic factors, such as population ageing, have a positive effect on public *HE* growth, but rather of a second order, when compared with other drivers, such as income level, technology, relative prices, and institutional settings (Medeiros and Schwierz 2013; Oliviera Martins and de la Maisonneuve 2006).

The focus of this paper is on the following question: Can public and private *HE* explain the health status variable like life expectancy at birth and infant mortality across different countries? This question is answered on a global scale with 195 countries for the years 1995–2014. Our analysis concerns the health impacts of public and private *HE*. We expect them to have different effects, as their provision and demand are not distributed uniformly within the population in each country. In addition, the nature and the quality of public health services differ significantly from those of private ones, especially in the poorest countries. Thus, the second question of interest is as follows: Do increased public and private expenditures in non-high-income countries bring more improvements in health status compared to high-income countries? This is the well-known income gradient hypothesis.

This paper is divided into five sections. In the following section, we provide a short review of material relevant to our questions and of some seminal papers. The third section elucidates the data used and describes the models applied and the methods involved. The fourth section presents the results. Subsequently, the paper ends with “Discussion and conclusions” section, which includes discussions and conclusions on the results.

## Health expenditures and health status

### Background

A major share of *HE* is publicly financed (i.e. through taxes or compulsory social insurance contributions), at least in OECD countries. While some believe that this and general income level may raise public *HE* because of additional demand resulting from a decrease in the net price of care, others suggest that the public financing of *HE* serves as a restraining factor. Research has shown that income increases are not the only primary drivers of the increase in the health share of the *GDP* (Gerdtham and Johnsson 2000). An interesting possibility is that institutional factors such as the spread of insurance coverage have not only directly encouraged spending but also have induced the adoption and diffusion of new medical technologies (Acemoglu and Finkelstein 2008). However, in high-income countries, macro-economic pressures on public budgets spill over to health budgets.

One approach to reducing the fraction of public financing is to increase out-of-pocket payments or private insurance. There are, however, major problems with this substitution. First, there is a limit as to how much out-of-pocket payments can be increased if the goal of equity is concerned. Second, private insurance as a means of financing poses a problem, because those with the highest potential expenditure also quite often have the lowest incomes. The public fraction of *HE* is highest in rich countries, which also often have the highest total expenditure. Private financing dominates in low-income countries, where direct out-of-pocket payments are more important than private insurance (Gerdtham and Johnsson 2000).

Focusing on the three major sources of *HE*—tax-financed spending, social security spending, and private spending—one sees clear differences in the structures of health care systems. South Asia is the region with the largest private sector share and virtually no reliance on social security systems. Africa and the Middle East rely heavily on private financing, but appear to have larger public tax-based (or externally supported) sources. In East Asia and the Pacific region, private spending is also high, but the public share has a significant portion in social security, driven almost exclusively by China (Hu and Ljungwall 2013; OECD 2014; Poullier et al. 2002; WHO 2014, 2015). The Americas also rely heavily on private financing, but somewhat less than other regions. It is only in Europe and partly in the OECD that health systems depend less on private financing and rely instead on significant shares of both social security and tax-based funding.

The estimates of public expenditure on health (*HE*_*PUB*_) range from as low as 7% to almost 100% of all health spending. The wealthier and healthier countries tend to rely more heavily on public sources of funds as a share of total spending. Generally, *HE*_*PUB*_ as a *share of total health spending* is poorly correlated with per capita *GDP*, even if the correlation is statistically different from zero (Ke et al. 2011; Poullier et al. 2002). However, once again, countries and regions vary significantly along this dimension. One way of looking at this wide range of public commitment to *HE* is to compare countries in groups with similar per capita income levels or health outcomes.

Although private health spending (*HE*_*PRIV*_) is overwhelmingly paid out-of-pocket, the share of private health insurance in total *HE* is insignificant in most countries. Prepaid private insurance accounts for more than 5% of *HE*_*PRIV*_ in only about one-third of the word’s countries. In those countries where private insurance has some significance, this averages only 26% of private spending, while private spending as a whole accounts only for an average of 10% of all health spending (Liang and Mirelman 2014; Poullier et al. 2002; WHO 2015). The bulk of private spending is paid out-of-pocket at the time of service. This out-of-pocket spending accounts for a much greater share of *HE* in relatively poor countries than in richer ones. The high level of out-of-pocket spending or very low public spending in some low- and middle-income countries stands out as one of the most troubling areas for public health policy.

There is a growing call for bilateral and multilateral agencies to increase their financial support to the health systems in low-income and high-disease-burden countries (e.g. Ebola epidemic in Western Africa in 2014–2015). There is also a growing concern among countries that provide grants and loans that their funds are targeted effectively to the populations with most need. However, most external funding goes to countries with large populations. In other cases, countries seem to be picked out for special assistance because they are recovering from war or dealing with severe hunger. Political attachments between particular countries also play a role in this context, but general, non-targeted aid seems to be unsuccessful (Deaton 2013).

Expenditure on health naturally depends on the number of people in need of health care. This is determined by factors such as population size and age composition. Expenditure is perceived to increase considerably at older ages, as older people often require costly medical treatment due to multi-morbidities and chronic illnesses. Improvements in life expectancy may therefore lead to increases in *HE* if not accompanied by improvements in general health status. The relation between life expectancy and *HE* is complicated by the fact that it is also influenced by the proximity to death, at least in the high-income countries.

### Econometric studies with *HE* effects on health outcomes

Improvements in human and health capital are critical catalysts to economic growth and development in the macroeconomic literature (López-Casasnovas et al. 2005). Good health improves not only individuals’ consumption and production in the short run, but also returns from investments in productive activities and education in the long run. At the macro level, investment in the health workforce and infrastructure is expected to improve health conditions and hence the health capital of the population. However, in many developing regions where resources are relatively scarce, *HE* has received less attention in government budgets. At the African regional level, Anyanwu and Erhijakpor (2007) found with a panel data fixed effect model that total *HE* was a significant contributor to health outcomes, with a 10% increase in total health care expenditure per capita resulting in a 21% and 22% decrease in under-five and infant mortality rates, respectively. Akinkugbe and Afeikhena (2006) also provided evidence that the effect of health care expenditure as a ratio of *GDP* on life expectancy, under-five mortality, and infant mortality was positive and significant in developing regions.

Filmer and Pritchett (1997) provided evidence that while health care spending impacted on child mortality, it was *not* the dominant driver of this health outcome. Factors such as education, technological change, and social capabilities have been identified by some researchers as major drivers of health outcomes rather than health care spending (Caldwell 1986; Easterly and Levine 1996; Lleras-Muney and Sherry 2008; Musgrove 1996). To understand why public spending on health has failed to have a strong effect on reducing mortality, Filmer and Pritchett (1999) stressed in their study that they were not suggesting that medical services are not (potentially) effective. They were not arguing that penicillin, immunizations, or oral rehydration therapy are ineffective as health interventions. But the impact of *HE*_*PUB*_ on health is much more complicated than the effectiveness of particular services purchased. For public spending to improve health cheaply (i.e. if money mattered), three things need to happen. Firstly, public spending is required to create effective health services. Secondly, the existence of those new public services is expected to change the total amount of effective health services consumed by the population. Thirdly, the additional services consumed must be cost-effective in improving health. If any one of these conditions is not met, the actual cost of services becomes high (Deaton 2013, chapter 9; Filmer and Pritchett 1999).

Partly contrary to these results, some studies claim to have found a clear significant impact of *HE*_*PUB*_ on health (Anand and Ravallion 1993; Baldacci et al. 2002; Bidani and Ravallion 1997; Jamison et al. 1996). For example, Bidani and Ravallion (1997) used a particular functional form to separate out the impacts of various variables on the poor and the non-poor. They found health status effects of *HE*_*PUB*_ spending for the poor but not for the non-poor. Their findings highlighted the importance of considering the incidence of the health benefits (i.e. some benefits helped best only the poor, so cuts without reallocation also fall on the poor). Note that Filmer and Pritchett (1999) also found that the impact on the poor versus the non-poor depended on the composition and efficacy of public spending.

One of the purposes of the study by Novignon et al. (2012) was to investigate the impact of total health care spending on various health outcomes after controlling for country-specific demographic structures and economic conditions. A differential analysis of public and private health care spending was also performed. The hypothesis was that there was no significant relationship between health spending and health outcomes in Sub-Saharan Africa. A further hypothesis was that there was no significant difference in the effects of public (*HE*_*PUB*_) and *HE*_*PRIV*_ on health outcomes. Results from the fixed and random effects models showed that a 1% increase in total *HE* (as % of *GDP*) was more likely to increase life expectancy at birth by approximately 0.6–0.7 years. Disintegrating the effect of total *HE* showed that a 1% increase in both *HE*_*PUB*_ and *HE*_*PRIV*_ significantly increased life expectancy at birth by about one and 0.4–0.5 years, respectively. Similar results were obtained for death rate (per 1000 people) and infant mortality rate (per 1000 live births).

For OECD countries, the *HE* effects on health are not directly comparable to the above low-income country results (see e.g. Barthold et al. 2014; Heijink et al. 2013; Jaba et al. 2014; Nixon and Ulmann 2006; van Baal et al. 2013). Note that private vs. public expenditure effects are analysed in very few papers. Only the papers by Cremieux et al. (2005), Lichtenberg (2000), and Or (2000) provide some information on this question. Cremieux et al. (2005) use data on Canadian provinces over the period 1975–1998. They focus on public and private spending on drugs with many additional variables with panel fixed effects regression methods. Results show that for life expectancy at birth, increases in drug spending and private spending effect are somewhat larger than public expenditure effect. Lichtenberg (2000) provides time series evidence from the US over the period 1960–1997 for life expectancy at birth in dynamic models where public and private expenditures predict life expectancy together with *GDP* and new molecular drug approvals. Public expenditure’s short- and long-term effects are statistically significant, but private effects are not precise, especially when lagged *GDP* is added in the model. Or (2000) uses similar methods as Cremieux et al. (2005) but explains premature death in 21 OECD countries in 1970–1992 with total *HE* and with share of public *HE* of total *HE* augmented with public health, environmental factors, and *GDP*. Note that Heijink et al. (2013) control for vast number variables and time trends in their analysis, but *HE* remains a significant determinant of avoidable mortality.

## Models, data, and methods

### Setup

The argument that income level—either personal or *GDP* per capita level—determines the health conditions of individuals and the population is profound in the health economics literature. However, the heterogeneity in health status between individuals or nations, even at the same income levels, requires a more detailed relationship between health conditions and specific expenditures targeted to promote health and care provision. The distinction between public and private expenditures here is important, since the former is likely a policy variable determined by the level of *GDPc* and the political agenda by the state and local public authorities, and the latter reflects the individual-level resources devoted to health care. Thus, both are endogenous variables in the long run. However, past findings suggest that the exogenous direct and delayed effects of *HE*_*PUB*_ on life expectancy and on infant mortality are positive and significant. Taking *HE*_*PRIV*_ as an exogenous variable is less warranted, as it is a form of derived demand (i.e. sickness and illness force people with short-run income constraints to put their money on *HE*).

Next, we propose dynamic panel data models to determine the levels of life expectancy at birth (*LE*) and infant mortality rate (*IM*), depending on $HE_{PRIV}$ and $HE_{PUB}$. We prefer the logarithmic forms of the variables in the following dynamic panel fixed effects (*FE*) model.

1 $$ \begin{aligned} lnLE_{it} & = \alpha_{0} + \alpha_{i} + \alpha_{1} lnLE_{i,t - 1} + \alpha_{2} lnHE_{PRIV,it} \\ &\quad +\,\alpha_{3} lnHE_{PUB,it} + \alpha_{4} lnPCR_{it} + \alpha_{5} lnRDE_{it} + \varepsilon_{i,1t} \\ lnIM_{it} & = \beta_{0} + \beta_{i} + \beta_{1} lnIM_{i,t - 1} + \beta_{2} lnHE_{PRIV,it}\\&\quad  +\, \beta_{3} lnHE_{PUB,it} + \beta_{4} lnPCR_{it} + \beta_{5} lnFS_{it} + \varepsilon_{i,2t} \\ \end{aligned} $$

In the first model, life expectancy is determined by private and public expenditures, and by exogenous variables of primary education rate (*PCR*) and level of R&D expenditures per capita (*RDE*). In the model for infant mortality, we replace *RDE* for food supply (*FS*). We stress that *HE* are direct means and resources to achieve good health and care among the population in the country, not the income level of the country as such. Thus, in the above models, the level of education, level of technology, and *FS* per capita refer generally (among many other similar variables) to the country’s development level that sustains life expectancy and lowers infant mortality.

One-period lagged health variables, *LE*_−1_ and *IM*_−1_, in models reflect the dynamics of health status (i.e. past health status determines the current one). Note, however, that both equations can be recursively solved for current and past values of other variables in the models and for the *starting values* of life expectancy and infant mortality (i.e. *LE*_*i*,0_ and *IM*_*i,*0_). These and other variables’ effects on current values of *LE*_*it*_ and *IM*_*it*_ are determined by the sizes of adjustment parameters $(\alpha_{1} \;{\text{and}}\;\beta_{1} )$. If they are close to but below one, the past variable values can still have large effects on current-level health status (see Eq. 3 below).

In general terms, the model captures more directly the income-driven health part of the bi-directional income–health relationship (Weil 2009, chapter 6). Income per capita, and other indicators of the living standard determine the health status of a country’s population. For example, if primary schooling is missing and the *FS* per capita is low, the income level of the country is typically low and the average health status is also low. Evidently, the so-called growth process has not started or it has halted because of missing factors that are important to sustain income generation. Although the needs for health care and medication are most urgent, the resources for them are sparse, even missing, or used elsewhere.

Our main argument is that, at least for poor countries, the resources devoted to public health provision—the $HE_{PUB}$—are more important for the population’s health status than the private expenditures. The reason for this stems from the large (income) inequalities prevailing in most poor countries supporting high incomes and *HE*_*PRIV*_ only for a small fraction of the population. The large population share of the poor can only get health benefits of public health care that is not exclusive.

### Data

Annual data from 195 countries in the years 1995–2014 were collected from different sources. The life expectancy variable (*LE*) (life expectancy at birth in total years) was collected from world development indicators (World Bank 2015). Additional data for life expectancy for some countries were taken from Gapminder (2015). The infant mortality rate (*IM*) (number of deaths of less than 1-year-old infants/1000 live births) was provided by the World Bank and WHO. Total, public, and private *HE* as  % shares of *GDP* (i.e. *HE*^*S*^, $HE_{PUB}^{S}$, and $HE_{PRIV}^{S}$) were taken from the Global Health Expenditure Database of the WHO (2015). Further health spending data for OECD countries were taken from OECD (2015) and derived for non-OECD countries with data on *GNI* per capita (formerly *GNP* per capita). *GNIc* is the gross national income, converted to U.S. dollars using the World Bank Atlas method, divided by the mid-year population (World Bank 2016). $HE_{PRIV}$ and $HE_{PUB}$ were derived as fractions of *GNIc* with $HE_{PUB}^{S}$ and $HE_{PRIV}^{S}$. The variable *PRC* (total of primary education completion rate as a  % of the relevant age group) is the  % of students completing the last year of primary school (World Bank 2015). R&D expenditures (*RDE*) were derived from R&D expenditure as a  % of *GDPc* (World Bank 2015). Finally, we obtained the variable *FS* (kilocalories per person per day) from the World Bank (2016).

### Country clusters and groups

In order to analyse effectively the public and private expenditure effects on life expectancy and infant mortality, we used the following country grouping strategy. We need different country clusters and groups to identify how public and private expenditure determines life expectancy and infant mortality that are quite heterogeneous across the sample countries and sample years. The *K*-means cluster method identified two clusters of countries with average country-specific growth rates of life expectancy in the sample period 1995–2014 (i.e. $\tfrac{1}{T - 1}\sum\nolimits_{t = 2}^{T} {\Delta lnLE_{it} }$). Table 1 reports the cluster mean values and number of cluster countries. We observe that in cluster 1 the mean growth rate of life expectancy was 3.5 times larger than in cluster 2. Typically, cluster 1 includes some of the poorest countries that have experienced significant health benefits from their care systems started in recent years.

**Table 1:** Clusters in average growth rate of life expectancy

	Cluster	
	1	2
Cluster mean	0.00872	0.00257
Number of countries	42	153

Next, the *K*-means method was also applied to growth rates of infant mortality rates (see Table 2). Due to the heterogeneous growth rates of infant mortality, the method proposed three clusters for average growth rates of infant mortality rates ($\tfrac{1}{T - 1}\sum\nolimits_{t = 2}^{T} {\Delta lnIM_{it} }$). Here cluster 3 comprises countries that belong to both *LE* growth clusters, that is, countries whose development process started before the sample period and their rapid progress in health status can also be seen in fast-declining infant mortality rates (e.g. China, Turkey, Brasil). Cluster 1 contains some of the poorest countries but also some developed countries that have already obtained a low level of infant mortality that is not declining anymore. Thus, most cluster countries here belong to life expectancy growth cluster 2. In infant mortality cluster 2, a typical country is a rich country (i.e. European country) with relatively low growth in life expectancy, but also some non-rich countries with a rapidly rising life expectancy (e.g. India).

**Table 2:** Clusters in average growth rate of infant mortality

	Cluster		
	1	2	3
Cluster mean	− 0.0125	− 0.0349	− 0.0615
Number of countries	56	100	39

Finally, we divided the countries into two groups based on their *average* level of *GNIc* during the sample period. If a country’s average *GNIc* level was below 2440 US$ during the sample years, it belonged to group 1 (77 countries, 39.5% of countries); countries with a level higher than 2440 US$ formed group 2 (118 countries, 60.5%). Note that in the sample, the mean income is 10085 US$ and the median is 3298 US$. Thus, 2440 US$ is close to 75% of the global median income in the years 1995–2014. This means that group 1 countries are globally the poorest countries.

### Summary statistics

Tables 3, 4, 5 provide detailed summary statistics in different clusters and income groups. In Table 3, clusters based on average life expectancy growth across the sample countries show that between low- and high-growth countries the difference between life expectancy is 11 years. Thus, during the sample period (1995–2014) the high level of life expectancy means less growth in life expectancy than at a lower level of life expectancy. The level of private and public *HE* per capita is 7–10 times larger in cluster 2 than in cluster 1.

**Table 3:** Summary statistics for life expectancy growth clusters

	*ΔlnLE*	*LE*	*HE_priv*	*HE_pub*
*CLUSTER 1*				
Mean	0.0081	60.638	39.718	60.906
SE of mean	0.0012	0.286	2.174	5.076
Standard deviation	0.035	8.287	63.016	147.119
CV	4.402	0.137	1.586	2.415
Median	0.0076	60.101	17.275	12.387
Sample size	798	840	840	840
*CLUSTER 2*				
Mean	0.0027	71.523	271.344	636.829
SE of mean	0.0002	0.141	8.285	23.621
Standard deviation	0.0145	7.815	458.309	1119.407
CV	5.297	0.109	1.689	1.757
Median	0.0027	73.601	98.058	173.977
Sample size	2907	3060	3060	3060

**Table 4:** Summary statistics for infant mortality clusters

	*ΔlnIM*	*IM*	*HE_priv*	*HE_pub*
*CLUSTER 1*				
Mean	− 0.0146	40.583	234.764	420.675
SE of mean	0.0006	0.954	18.083	26.263
Standard deviation	0.0247	31.924	605.189	882.296
CV	− 1.482	0.786	2.577	2.048
Median	− 0.0161	31.551	47.969	118.399
Sample size	1064	1120	1120	1120
*CLUSTER 2*				
Mean	− 0.0341	33.754	219.487	592.067
SE of mean	0.0006	0.618	7.410	25.985
Standard deviation	0.0247	30.491	331.392	1162.122
CV	− 0.721	0.903	1.510	1.963
Median	− 0.0331	22.610	57.637	83.022
Sample size	1900	2000	2000	2000
*CLUSTER 3*				
Mean	− 0.0592	26.900	207.348	427.396
SE of mean	0.0011	1.108	9.205	22.888
Standard deviation	0.0287	28.449	257.096	799.483
CV	− 0.485	1.057	1.239	1.823
Median	− 0.0579	15.301	116.807	160.625
Sample size	741	780	780	780

**Table 5:** Summary statistics for *GNI per capita* level groups

	*ΔlnLE*	*LE*	*ΔlnIM*	*IM*	*HE_priv*	*HE_pub*
*GROUP 1*						
Mean	0.0052	61.846	− 0.0333	58.871	27.097	29.181
SE of mean	0.0008	0.197	0.0007	0.749	0.836	1.376
Standard deviation	0.0311	7.736	0.0277	29.412	32.827	53.021
CV	5.979	0.125	− 0.031	0.499	1.211	1.851
Median	0.0045	62.010	− 0.0315	56.851	15.741	14.050
Sample size	1463	1540	1463	1540	1540	1540
*GROUP 2*						
Mean	0.0030	73.964	− 0.0339	18.340	348.282	828.355
SE of mean	0.0020	0.129	0.0006	0.392	10.237	24.966
Standard deviation	0.0095	6.299	0.0300	19.033	497.315	1212.87
CV	3.162	0.085	− 0.887	1.037	1.428	1.464
Median	0.0027	75.001	− 0.0309	12.701	169.356	309.718
Sample size	2242	2360	22420	2360	2360	2360

The distributions of expenditures are skewed towards low values corresponding to *GNIc* distributions among the global countries. Note also the large standard deviation (and *CV*) values showing large heterogeneity, especially in *ΔlnLE* and expenditure observations.

Similar remarks are valid for the infant mortality growth clusters, but now we also observe (see Table 4), that the levels of *HE* across the clusters are not as large as in the above life expectancy clusters. Particularly, the level of *HE*_*PRIV*_ does not vary across the *ΔlnIM* clusters and levels of *IM.* Thus, infant mortality seems to be independent of *HE*_*PRIV*_. However, the level of *IM* clearly determines the speed of its decline (i.e. the lower the level of infant mortality rate is, the larger the decrease is). Note also that *IM* distributions are skewed towards low values.

Table 5 provides summary statistics for *GNI* per capita level groups. The most interesting result is that the rate of decrease of infant mortality (*ΔlnIM*) is almost the same in both *GNIc* groups, albeit there is a huge difference between the *levels* of infant mortality (*IM*). The difference between life expectancy (*LE*) is 12 years, but in poor countries the growth rate of life expectancy is almost two times greater than in non-poor countries. However, a huge difference remains between the country groups in levels of *HE*.

Generally, these findings with respect to our dynamic panel data models mean that we do not expect much success in infant mortality modelling, as the variable seems to be insensitive to the level of private expenditure. However, the large within heterogeneity in clusters and income groups masked by the above location statistics need a country-level fixed effect (*FE*) modelling approach that can provide some valuable results across the cluster countries.

### Dynamic panel data models

Consider the following dynamic fixed (*FE*) or random (*RE*) effect model (for more details, see Pesaran 2015, chapters 26–27):

2 $$ y_{it} = \alpha_{i} + \lambda y_{i,t - 1} + \beta^{{\prime }} x_{it} + \mu_{it} ,\quad i = 1,2,{ \ldots },N\;{\text{and}}\;t = 1,2,{ \ldots },T. $$

Typically, regressors, $\varvec{x}_{it} ,$ are assumed to be *strictly exogenous* (i.e. $E[\mu_{it} |\varvec{x}_{it} ] = 0$) for all *i* and *t*. However, the assumption of strict exogeneity is not valid by construction for lagged dependent variable $y_{i,t - 1}$, since even if we assume that $E[\mu_{it} \alpha_{i} ] = E[\mu_{it} y_{i0} ] = 0,$ the *FE/RE* demeaning term $E[\mu_{it} \bar{y}_{i, - 1} ] \ne 0$ will not vanish for short panels. In the process without regressors $\varvec{x}_{it} ,$ this will cause bias for the *FE* or *RE* estimators of $\lambda$ with its size depending on the true value of $|\lambda | < 1$ and the length of panels (Nickell 1981; Pesaran 2015, p. 679)

$$ \mathop {plim}\limits_{N \to \infty } (\hat{\lambda }_{FE/RE} - \lambda ) = - \frac{(1 + \lambda )}{T} + O(T^{ - 2} ). $$

The bias is order of 1*/T* and vanishes when $T \to \infty .$ For example, when λ is close to 1 (the non-stationary case) and *T *=20, the bias is close to − 0.1. Note that if regressors $\varvec{x}_{it}$ are included in the model, the size of bias for λ and ***β*** depends on the correlation between $y_{i,t - 1}$ and $\varvec{x}_{it}$. If regressors $\varvec{x}_{it}$ are only weakly exogenous (i.e. allowing for feedbacks from $\mu_{i,t - 1}$) or if they are endogenous variables, the *FE/RE* bias for ***β*** is still present, even if no lagged dependent variable is found in the model.

The generic problems of the above dynamic panel model can be seen when we solve for $y_{it}$ recursively from the initial values of $y_{i0}$

3 $$ y_{it} = \lambda^{t} y_{i0} + \sum\nolimits_{j = 0}^{t} {\lambda^{j} \beta^{{\prime }} x_{i,t - j} + \frac{{1 - \lambda^{t} }}{1 - \lambda }\alpha_{i} + \sum\nolimits_{j = 0}^{t - 1} {\lambda^{j} \mu_{i,t - j} } } ,\quad t = 1,2,{ \ldots },T $$

When $\lambda$ is close to one, initial values $y_{i0}$ and unit-specific effects $\alpha_{i}$ have large and permanent effects on the $y_{it}$ observations determining the properties of dynamic panel data model estimators. As the process for $y_{i,t - 1}$ has a similar presentation, we obtain, abstracting from terms for regressors and errors:

$$ y_{it} - y_{i,t - 1} = (\lambda^{t} - \lambda^{t - 1} )y_{i0} + \lambda^{t - 2} \alpha_{i} . $$

This shows that initial effects, but not necessarily the unit-specific $\alpha_{i}$ effects, have a small role in determining the one-period differenced values of $y_{it}$. Subsequently, the following difference model has also been popular to eliminate the unit-specific effects on $\lambda {\text{ and }}\varvec{\beta}$ estimates:

4 $$ \Delta y_{it} = \lambda \Delta y_{i,t - 1} +\varvec{\beta}^{{\prime }} \Delta \varvec{x}_{it} + \Delta \mu_{it} . $$

However, this will not solve the (OLS) estimation problems for the model parameters, since

$$ E[\Delta y_{i,t - 1} \Delta \mu_{it} ] = E[\lambda \Delta \mu_{i,t - 1} \Delta \mu_{it} ] \ne 0. $$

$$ {\text{Because}}\;{\text{of}}\;E[\Delta \mu_{i,t - s} \Delta \mu_{it} ] = \left\{ {\begin{array}{*{20}l} {2\sigma_{u}^{2} ,} \hfill & {{\text{for}}\;s = 0} \hfill \\ { - \sigma_{u}^{2} ,} \hfill & {{\text{for}}\;s = 1} \hfill \\ {0,} \hfill & {{\text{for}}\;s > 1} \hfill \\ \end{array} } \right. $$

we need at least two-period lagged values of $$ y_{i,t - j} {\text{ and }}\Delta y_{i,t - j} $$$$ (j \ge 2) $$ that do not correlate with $$ \Delta \mu_{it} $$ (but correlate with $$ \Delta y_{i,t - 1} $$). We can use them as instruments for $$ \Delta y_{i,t - 1} $$ as long as $$ \lambda < 1, $$ but as $$ \lambda \to 1 $$, we face the weak instrument problem for $$ y_{i,t - 2} $$ because $$ E[y_{i,t - 2} ,\Delta y_{i,t - 1} ] $$ depends on the size of λ (for more details, see Pesaran 2015, p. 682).

The short panel bias of *FE/RE* and the efficiency problem of the *IV* approach for the first difference model started the search for *IV/GMM*-type estimators, leading to consistent and more efficient estimators like *GMM* estimators by Arellano and Bond (1991), Ahn and Schmidt (1995), and Blundell and Bond (1998). These surprisingly popular methods are extremely complex estimators, which are unbiased and efficient only when no residual serial correlation is found, the dynamic lag order of the model is correctly specified, we have strictly exogenous regressors, no correlation is found between explanatory variables and unit-specific effects $a_{i}$, errors are homoscedastic, the sample length is small (i.e. $T/n \to 0$ convergence), low autocorrelation is present in endogenous series, and the problem of weak or too many instruments is not present (see e.g. Dang et al. 2015; Gouriéroux et al. 2010; Hahn et al. 2007; Kiviet et al. 2017).

Empirical drawbacks of the *IV/GMM* agenda have led to a large group of alternative estimators that have tried in several different ways to correct for 1*/T* time series bias. Chudik and Pesaran (2015) divide this literature into the following broad categories: (i) analytical corrections based on an asymptotic bias formula (Bruno 2005; Bun 2003; Bun and Carree 2005, 2006; Bun and Kiviet 2003; Hahn and Kuersteiner 2002; Hahn and Moon 2006; Kiviet 1995, 1999), (ii) bootstrap and simulation-based bias corrections (Everaert and Ponzi 2007; Phillips and Sul 2003, 2007), and (iii) other methods, including jackknife bias corrections (Dhaene and Jochmans 2012) and the recursive mean adjustment correction procedures (So and Shin 1999). In addition, some methods have been proposed on long differences (Hahn et al. 2007; Han and Phillips 2013; Han et al. 2014), on forward filtering (Keane and Runkle 1992; Keane and Neal 2016; Pesaran 2015, chapter 27.2), and on the transformed likelihood method (Hayakawa and Pesaran 2015; Pesaran 2015, chapter 27.6).

In the following, we take methods that are planned to be robust enough against near unit-root case and avoid strict exogeneity assumption. This means that we use a long difference *IV* method, *LD*_*IV*_, proposed by Hahn et al. (2007) as well as the Keane–Runkle estimator (1992), which allows for predetermined variables as instruments.

The *LD*_*IV*_ technique uses long differencing, i.e. $\Delta_{k} y_{t} = y_{t} - y_{t - k}$ with $k = 2,3,4, \ldots$, instead of first differencing and iterated two-stage least square (*2SLS*) in estimating persistent dynamic models with a short time dimension. The *LD*_*IV*_ estimator uses lagged levels of the regressors (including $y_{i,t - k - 1}$) and the residuals as instruments. The setup for the model is (Hahn et al. 2007, pp. 586–587; Huang and Ritter 2009, p. 269):

5 $$ \Delta_{k} y_{it} = \lambda \Delta_{k} y_{it - 1} +\varvec{\beta}^{{\prime }} \Delta_{k} \varvec{x}_{it} + \Delta_{k} \mu_{it} $$

where we can use $y_{i,t - k - 1} ,\varvec{x}_{i,t - k}$ (if strictly exogenous or predetermined) as instrument variables. After obtaining *2SLS* estimates for model 5 we calculate the residuals

$$ y_{i,t - 1} - \hat{\lambda }y_{i,t - 2} - \hat{\varvec{\beta }}^{{\prime }} \varvec{x}_{i,t - 1} , \ldots ,\;{\text{and}}\;y_{i,t - k} - \hat{\lambda }y_{i,t - k - 1} - \hat{\varvec{\beta }}^{{\prime }} \varvec{x}_{i,t - k} . $$

Next we use these as additional instrumental variables with $y_{i,t - k - 1} ,\varvec{x}_{i,t - k}$ to estimate (5) once again. This is the first iteration. Next new *2SLS* estimations are then further iterated via the new results. Typically, less than five iterations are sufficient for convergence.

The Keane and Runkle (1992) estimator (*KR*_*PRE*_) uses the idea of forward filtering or decomposition from the time-series literature to improve the efficiency of the estimates when the error contains some form of serial correlation. Under Cholesky transformation, the orthogonality conditions implied by predetermination are maintained (Keane and Neal 2016). In practice, a key feature of the approach is to use only one or two lags of the predetermined variables as instruments rather than all available lags back to the first period like in complex *GMM* estimation. Keane and Runkle assume that $\varvec{x}_{it}$ are predetermined, in the sense that $E[\varvec{x}_{is} \mu_{it} ] = 0$, for *t *≥* s*. This is a natural approach in this context where public and private *HE* in current and previous periods drive life expectancy but not necessarily vice versa. However, life expectancy targets or its unobserved determinants in coming periods *t *+* i* (*i *=1,2,…) will affect public and private *HE* in the future (i.e. $E[\varvec{x}_{ir} \mu_{it} ] \ne 0$, for *r *>* t*). Note that in the first difference model $\varvec{x}_{it}$ is correlated with $\mu_{i,t - 1}$ because $\varvec{x}_{it}$ is predetermined but not strictly exogenous. However, $y_{i,t - 2} \;{\text{and}}\;\varvec{x}_{i,t - 1}$ are now valid instruments.

In the Keane–Runkle method, the model 2 has a general covariance specification for $$ v_{it} = \alpha_{i} + \mu_{it} $$. That is,$$ E[\varvec{vv}'] = \varvec{I}_{N} \otimes {\varvec{\Sigma}} $$, where $$ \varvec{v} $$ is a stacked $$ NT \times 1 $$ vector of $$ \varvec{v}_{i \cdot } = (v_{i1} ,v_{i2} ,{ \ldots },v_{iT} )^{{\prime }} $$ and $$ {\varvec{\Sigma}} = E[\varvec{v}_{i \cdot } \varvec{v}_{i \cdot }^{{\prime }} ] $$. To implement the *KR*_*PRE*_ estimator, we need an estimate for $$ {\varvec{\Sigma}} $$. It is obtained from consistent preliminary *2SLS/IV* estimation of model 2 using the instruments $$ \varvec{Z} $$ to obtain the *2SLS/IV* residuals $$ \hat{\varvec{v}}_{i \cdot }^{{}} $$ and $$ {{\hat{\boldsymbol{\Sigma}}}} = \tfrac{1}{N}\sum\nolimits_{i =
1}^{N} {\hat{\varvec{v}}_{i \cdot }^{{\prime }} } hat{\varvec{v}}_{i
\cdot}. $$ Note that a similar two-step procedure can be applied also for difference model 4.

## Estimation results

### Life expectancy model

Tables 6 and 7 provide the dynamic panel data model estimation results for *lnLE* based on standard *FE*, weighted *FE*_*W/TR*_ with trend, *LD*_*IV*_, and *KR*_*PRE*_ estimation methods. These methods have validity in this context, since in preliminary data analysis the panel data non-stationarity tests (not reported) supported the stationarity alternative. The *FE* methods assume that all explanatory variables are strictly exogenous, but in *LD*_*IV*_ and *KR*_*PRE*_ this erroneous assumption is noticed (i.e. lagged *lnLE* is not exogenous), and we need *IV* methods to overcome this problem. In addition, in *KR*_*PRE*_ we use a less restricted approach on variables *lnLE*_−1_, *lnHE*_*PRIV*_, and *lnHE*_*PUB*_ when we assume that they are predetermined with periods $t \ge s.$ Thus, the *FE* approach is expected to be biased in short panels with 1*/T* rate. However, using a sample length of *T *=19, its role is less important than the evident sample heterogeneity and trending behavior of life expectancy panels. To obtain greater robustness in estimation, we use trend variable and cross-section weights in *FE*_*W/TR*_ estimation (i.e. we estimate the model with a feasible *GLS* specification, assuming the presence of cross-section heteroscedasticity). This has a large effect on the point estimates of model parameters, but their qualitative effects remain the same as in standard *FE* estimation. In the following tables below 10% level *p* values with coefficient estimate values are marked with bold font. In the high life expectancy growth rate cluster (cluster 1), *HE*_*PRIV*_ have imprecise, statistically non-significant effects on life expectancy, but *HE*_*PUB*_ and primary education rate (*lnPCR*) predict it with correct signs with 10% level significance. In the low life expectancy growth rate cluster (cluster 2), private expenditures also have some significance along with R&D expenditures. Note that education effects on life expectancy are generally larger than *HE* or R&D effects. Note that *DW* values refer to a Durbin-Watson type test statistics calculated on the stacked set of pooled model residuals. Thus the reported *DW* values are indicative, i.e. when the values are close to 2 we take it as a sign of appropriate model lag choice.

**Table 6:** *lnLE* models with *ΔlnLE* clusters (*p* values in parentheses)

Cluster 1 N = 42, T = 19	FE^1^	FE_W/TR_^1^	LD_IV_^2,3^	KR_PRE_^4^
*C*	**1.499** **(0.053)**	**0.539** **(0.001)**	–	**2.800** **(0.000)**
*lnLE*_*−*1_	**0.597** **(0.004)**	**0.862** **(0.000)**	**0.597** **(0.029)**	**0.194** **(0.001)**
*lnHE_priv*	0.006 (0.283)	− 0.001 (0.248)	0.003 (0.477)	− **0.049** **(0.012)**
*lnHE_pub*	**0.005** **(0.030)**	**0.001** **(0.073)**	0.005 (0.250)	**0.061** **(0.000)**
*lnPCR*	**0.028** **(0.094)**	**0.005** **(0.001)**	0.021 (0.205)	**0.092** **(0.001)**
*lnRDE*	**0.009** **(0.077)**	0.0006 (0.194)	0.011 (0.147)	− 0.002 (0.867)
*DW value*	2.44	2.36	–	–

Cluster 2 N = 153, T = 19	FE^1^	FE_W/TR_^1^	LD^2,3^	KR_PRE_^5^
*C*	**1.089** **(0.024)**	**0.512** **(0.000)**	–	**3.381** **(0.000)**
*lnLE*_−1_	**0.727** **(0.000)**	**0.875** **(0.000)**	**0.709** **(0.001)**	**0.122** **(0.036)**
*lnHE_priv*	0.002 (0.111)	**0.0007** **(0.000)**	0.0024 (0.103)	**0.012** **(0.000)**
*lnHE_pub*	**0.0004** **(0.005)**	**0.0004** **(0.006)**	**0.0025** **(0.002)**	**0.009** **(0.001)**
*lnPCR*	**0.008** **(0.002)**	**0.003** **(0.000)**	0.009 (0.158)	**0.052** **(0.000)**
*lnRDE*	**0.002** **(0.007)**	**0.0004** **(0.000)**	0.018 (0.132)	**0.004** **(0.029)**
*DW* value	1.63	1.73	–	**–**

^1^SEs calculated with White’s cross-section method

^2^SEs adjusted for cross-section clusters

^3^Instruments: lnLE(− 6), lnHE_priv(− 5), lnHE_pub(− 5), lnPCR(− 5), lnRDE(− 5), res(− 1 to − 4)

^4^Instruments: ΔlnLE(− 1), ΔlnHE_priv(− 1 to − 2), ΔlnHE_pub(− 1 to − 2), lnPCR, lnRDE, constant

^5^Instruments: ΔlnLE(− 1), lnHE_priv(− 1 to − 2), lnHE_pub(− 1 to − 2), lnPCR, lnRDE, constant

**Table 7:** *lnLE* models with *GNIPc* groups (*p* values in parentheses)

Group 1 N = 77, T = 19	FE^1^	FE_W/TR_^1^	LD_IV_^2,3^	KR_PRE_^5^
*C*	**1.309** **(0.022)**	**0.503** **(0.000)**	–	0.013 (0.122)
*lnLE*_−1_	**0.655** **(0.000)**	**0.872** **(0.000)**	**0.617** **(0.001)**	0.025 (0.532)
*lnHE_priv*	**0.004** **(0.029)**	− 0.0001 (0.177)	0.003 (0.212)	**0.043** **(0.000)**
*lnHE_pub*	**0.004** **(0.014)**	**0.001** **(0.009)**	0.004 (0.260)	**0.014** **(0.065)**
*lnPCR*	**0.023** **(0.019)**	**0.006** **(0.000)**	**0.021** **(0.060)**	**0.104** **(0.001)**
*lnRDE*	**0.006** **(0.028)**	**0.001** **(0.000)**	**0.006** **(0.068)**	**0.012** **(0.044)**
*DW* value	2.42	1.84	**–**	**–**

Group 2 N = 118, T = 19	FE^1^	FE_W/TR_^1^	LD_IV_^2,4^	KR_PRE_^6^
*C*	**0.796** **(0.000**	**0.426** **(0.000)**	–	− **0.021** **(0.032)**
*lnLE*_−1_	**0.806** **(0.000)**	**0.899** **(0.000)**	**0.781** **(0.000)**	**0.746** **(0.000)**
*lnHE_priv*	0.001 (0.152)	**0.0005** **(0.004)**	0.021 (0.351)	**0.012** **(0.033)**
*lnHE_pub*	**0.005** **(0.01)**	**0.0006** **(0.003)**	**0.005** **(0.026)**	**0.063** **(0.061)**
*lnPCR*	− 0.000 (0.970)	0.000 (0.671)	− 0.002 (0.897)	− 0.026 (0.152)
*lnRDE*	**0.002** **(0.000)**	**0.0003** **(0.001)**	**0.0014** **(0.001)**	− **0.024** **(0.069)**
*DW* value	1.84	1.69	**–**	**–**

^1^SEs calculated with White’s cross-section method

^2^SEs adjusted for cross-section clusters

^3^Instruments: lnLE(− 6), lnHE_priv(− 5), lnHE_pub(− 5), lnPCR(− 5), lnRDE(− 5), res(− 1 to − 4)

^4^Instruments: lnLE(− 16), lnHE_priv(− 15), lnHE_pub(− 15), lnPCR(− 15), lnRDE(− 15), res(− 1 to − 14)

^5^Instruments: ΔlnLE(− 1), lnHE_priv(− 1 to − 2), lnHE_pub(− 1 to − 2), lnPCR, lnRDE, constant

^6^Instruments: ΔlnLE(− 1), ΔlnHE_priv(− 1), ΔlnHE_pub(− 1), lnPCR, lnRDE, constant

Results with the *LD*_*IV*_ method are not promising. A search over a suitable difference length provided a five-period difference approach. Models with a trend variable were also considered, but they provided non-significant and partly wrongly signed estimates. Results with the *KR*_*PRE*_ method were quite different from the above-mentioned ones. This method seems to scale down the adjustment coefficient but provides larger point estimates for other model parameters. Note that *lnHE*_*PRIV*_ gets a negative coefficient estimate for cluster 1. *SE*s of *KR*_*PRE*_ estimates are not corrected for possible heteroscedasticity. Also, an instrument validity test is needed for *LD*_*IV*_ and *KR*_*PRE*_ estimations before their full validity can be evaluated. We do not report any model diagnostic values like *R*^2^ or *F*-test and residual diagnostics except *DW* values for *FE* models, as the different model estimators are based on different methods and provide statistics that are not comparable.

Table 7 provides the *lnLE* model estimates in *GNIc*-level groups. Results with *FE* methods are comparable with the results above. *HE*_*PUB*_ effects are positive and significant on life expectancy, but the size of the effects is less than for primary education. Surprisingly, the education effects are non-significant for non-poor countries. Results with the *LD*_*IV*_ method are close to standard *FE* results, but non-significant *HE* effects are obtained. *KR*_*PRE*_ estimation results are once again in their own category in income level group 1. This depends partly on the demeaning of variables that provided the best results. All but adjustment coefficients are significant, and the rest are comparable with *FE* and *LD* coefficients when these are solved for long-run presentation. However, for income group 2, although estimated with demeaned data, results are quite different. In general, these results—and many others not represented here—show that the *KR*_*PRE*_ method has some stability and robustness problems.

### Infant mortality model

These problems are clearly evident for infant mortality model estimation, as the country-level time series have *AR*(2) presentation more likely than *AR*(1) models. Although the *KR*_*PRE*_ method needs consistent *IV* estimation in the first stage, which runs easily into problems when the adjustment coefficient sum is close to one, the second-stage estimation will not correct these but also runs into problems. For these reasons, we did not provide results on *lnIM* models with *ΔlnIM* clusters and take a critical stance on *KR*_*PRE*_ estimation in *ΔlnIM* clusters.

Table 8 provides *FE* and *LD*_*IV*_ results for *lnIM* models with *ΔlnIM* clusters. We observe that *LD*_*IV*_ based on a long difference works with *AR*(1) presentation. Estimation results show that increases in *HE*_*PUB*_ reduce infant mortality, but the *FS* has an infant-reducing effect only in cluster 3, where the decline in infant mortality is largest. Surprisingly, *HE*_*PRIV*_ have an increasing infant mortality effect in this cluster. In other clusters, the effects are imprecise.

**Table 8:** *lnIM* models with *ΔlnIM* clusters (*p* values in parentheses)

Cluster 1 N = 56, T = 18	FE^1^	FE_W/TR_^1^	LD_IV_^2,3^
*C*	− 0.106 (0.475)	**0.060** **(0.047)**	–
*lnIM*_−1_	**1.501** **(0.000)**	**1.698** **(0.000)**	**0.977** **(0.001)**
*lnIM*_−2_	− **0.542** **(0.000)**	− **0.739** **(0.000)**	–
*lnHE_pub*	− **0.003** **(0.0002)**	− 0.0005 (0.254)	0.002 (0.301)
*lnHE_priv*	− **0.005** **(0.000)**	− **0.0007** **(0.017)**	− **0.004** **(0.018)**
*lnPCR*	− **0.006** **(0.094)**	− 0.0007 (0.336)	0.004 (0.567)
*lnFS*	**0.036** **(0.032)**	**0.011** **(0.003)**	**0.046** **(0.028)**
*DW* value	**2.35**	**2.44**	**–**

Cluster 2 N = 100, T = 18	FE^1^	FE_W/TR_^1^	LD_IV_^2,4^
*C*	**0.210** **(0.021)**	**0.126** **(0.000)**	–
*lnIM*_−1_	**1.079** **(0.000)**	**1.491** **(0.000)**	**0.975** **(0.001)**
*lnIM*_−2_	− 0.119 (0.492)	− **0.532** **(0.000)**	–
*lnHE_priv*	− 0.003 (0.299)	− 0.0008 (0.101)	− 0.004 (0.345)
*lnHE_pub*	− **0.006** **(0.001)**	− **0.0007** **(0.046)**	− 0.004 (0.291)
*lnPCR*	− **0.020** **(0.005)**	− **0.004** **(0.005)**	− **0.018** **(0.031)**
*lnFS*	0.0009 (0.941)	0.002 (0.313)	0.003 (0.108)
*DW* value	2.20	2.47	–

Cluster 3 N = 39, T = 18	FE^1^	FE_W/TR_^1^	LD_IV_^2,5^
*C*	**0.444** **(0.019)**	**0.186** **(0.000)**	–
*lnIM*_−1_	**1.215** **(0.000)**	**1.657** **(0.061)**	**1.012** **(0.000)**
*lnIM*_−2_	− **0.225** **(0.088)**	− **0.677** **(0.000)**	–
*lnHE_priv*	**0.005** **(0.081)**	**0.002** **(0.078)**	**0.016** **(0.020)**
*lnHE_pub*	− **0.007** **(0.005)**	− **0.002** **(0.070)**	− 0.006 (0.292)
*lnPCR*	− **0.034** **(0.000)**	− 0.007 (0.126)	− **0.050** **(0.000)**
*lnFS*	− **0.038** **(0.075)**	− **0.014** **(0.0006)**	− 0.038 (0.266)
*DW* value	2.30	2.49	–

^1^SEs calculated with White’s cross-section method

^2^SEs adjusted for cross-section clusters

^3^Instruments: lnIM(− 4), lnHE_priv(− 3), lnHE_pub(− 3), lnPCR(− 3), lnFS(− 3), res(− 1 to − 2). Model includes a trend

^4^Instruments: lnIM(− 16), lnHE_priv(− 15), lnHE_pub(− 15), lnPCR(− 15), lnFS(− 15, res(− 1 to − 14)

^5^Instruments: lnIM(− 6), lnHE_priv(− 5), lnHE_pub(− 5), lnPCR(− 5), lnFS(− 5), res(− 1 to − 4)

Table 9 provides results on *lnIM* models with *GNIc* groups and also includes some *KR*_*PRE*_ estimation results. Generally, results in Table 9 are not favorable for *HE* variables. Public expenditures reduce infant mortality in non-poor countries, but similar effects are not found for private expenditures in either income group. Education effects reducing infant mortality are clearly present in Table 9 compared to Table 8. *FS* effects are mortality-reducing in poor countries.

**Table 9:** *lnIM* models with *GNIc* groups (*p* values in parentheses)

Group 1 N = 77, T = 18	FE^1^	FE_W/TR_^1^	LD_IV_^2,3^	KR_PRE_^5^
*C*	**0.458** **(0.004)**	**0.098** **(0.001)**	–	− **0.004** **(0.049)**
*lnIM*_−1_	**0.970** **(0.000)**	**1.610** **(0.000)**	**0.980** **(0.000)**	**0.819** **(0.000)**
*lnIM*_−2_	0.0134 (0.946)	− **0.612** **(0.000)**	–	–
*lnHE_priv*	− 0.002 (0.591)	− 0.0005 (0.247)	**0.007** **(0.014)**	− 0.026 (0.247)
*lnHE_pub*	− 0.001 (0.401)	**0.0004** **(0.090)**	− 0.001 (0.767)	− 0.007 (0.415)
*lnPCR*	− **0.018** **(0.0016)**	− **0.0019** **(0.087)**	**v0.088** **(0.092)**	− 0.003 (0.415)
*lnFS*	− **0.045** **(0.009)**	− **0.004** **(0.040)**	− **0.043** **(0.069)**	− 0.431 (0.122)
*DW* value	2.14	2.53	–	–

Group 2 N = 118, T = 18	FE^1^	FE_W/TR_^1^	LD_IV_^2,4^	KR_PRE_^6^
*C*	− 0.044 (0.487)	0.013 (0.584)	–	− **0.002** **(0.034)**
*lnIM*_−1_	**1.397** **(0.000)**	**1.615** **(0.000)**	**0.986** **(0.000)**	**0.889** **(0.000)**
*lnIM*_−2_	− **0.417** **(0.000)**	− **0.635** **(0.000)**	–	–
*lnHE_priv*	0.0001 (0.927)	− 0.0008 (0.105)	0.001 (0.878)	− 0.002 (0.834)
*lnHE_pub*	− **0.006** **(0.0005)**	− 0.0007 (0.352)	− **0.009** **(0.063)**	− **0.019** **(0.045)**
*lnPCR*	− **0.010** **(0.073)**	0.0014 (0.381)	− **0.035** **(0.081)**	0.001 (0.885)
*lnFS*	**0.019** **(0.018)**	0.004 (0.201)	**0.059** **(0.035)**	**0.026** **(0.055)**
*DW* value	2.42	2.43	–	–

^1^SEs calculated with White’s cross-section method

^2^SEs adjusted for cross-section clusters

^3^Instruments: lnIM(− 6), lnHE_priv(− 5), lnHE_pub(− 5), lnPCR(− 5), lnFS(− 5), res(− 1 to − 4). Model includes a trend

^4^Instruments: lnIM(− 16), lnHE_priv(− 15), lnHE_pub(− 15), lnPCR(− 15), lnFS(− 15), res(− 1 to − 14). Model includes a trend

^5^Instruments: lnIM(− 2), lnHE_priv(− 1 to − 2), lnHE_pub(− 1 to − 2), lnPCR, lnRDE, constant, (difference model)

^6^Instruments: lnIM(− 2), lnHE_priv(− 1 to − 2), lnHE_pub(− 1 to − 2), lnPCR, lnRDE, constant, (difference model)

Generally, we observe that different methods provide quite varying results on point estimates, but clear qualitative accordance is found in the signs of coefficient estimates across the estimation methods. The *LD*_*IV*_ method provides results that are comparable to *FE* results, but *KR*_*PRE*_ produced results that are less clear. Nevertheless, we calculate long-run estimates or elasticities for *HE* variables from estimation results at 10% or below the significance level in the above tables. Note that we did not calculate *SE*s of the long-run estimates. That can be done with a delta method. Table 10 provides the sum-up. It shows that across the different data configurations and model estimations, 22 *HE*_*PUB*_ coefficient estimates from 36 possible ones were significant and their mean of long-run elasticities is 0.0364 for life expectancy and − 0.223 for infant mortality. Thus, if public expenditures increase by 10%, they will increase life expectancy by 0.36% and reduce infant mortality by 2.31%. For private expenditures, we find 0.23% and − 0.83% mean elasticities for a much smaller number of 10%-level significant estimates.

**Table 10:** Long-run elasticities

Variables	Number of significant estimates	LR-elasticity estimate range	Mean LR-elasticity
*lnHE_priv *→* lnLE*	7	[− 0.0607, 0.0560]	0.0229
*lnHE_pub *→* lnLE*	11	[0.00144, 0.248]	0.0364
*lnHE_priv *→* lnIM*	5	[− 1.333, 0.50]	− 0.0833
*lnHE_pub *→* lnIM*	11	[− 0.6435, 0.0759]	− 0.2231

These estimates and related ones in the literature imply that *HE*_*PUB*_ are more effective to improve health status than private expenditures for a large number of countries. However, we cannot say that this is also true for the poorest countries in our sample (see Table 6: cluster 1, and Tables 7 and 9: *GNIc* group 1), since we have too few estimates to prove this. Some results with a larger data set (34 OECD countries in the years 1970–2012) and different methods have shown that public expenditure effects in non-poor countries dominate the private ones (see e.g. Linden and Ray 2017). At the moment, partly because first-class data on life expectancy and infant mortality from the poorest countries are not available, the question with poor countries is not fully answerable.

## Discussion and conclusions

The *HE* effects on life expectancy at birth and infant mortality were analyzed with dynamic panel data models for 195 countries in the years 1995–2014. The target questions of ‘Can public and private *HE* explain the health status variables like life expectancy at birth and infant mortality across different countries?’ and ‘Do increased public and private expenditures in non-high-income countries bring more improvements in health status compared to high-income countries?’ were analyzed with country clusters sensitive to life expectancy growth rates and rates of decrease in infant mortality, and with low and high *GNI* per capita levels.

The first question gets an affirmative answer, and obtained results show that *HE*_*PUB*_ are generally more health-promoting than private expenditures. However, either of the *HE* effects is not as large as primary education effects. We were not able to give a positive answer to the second question. In country clusters and groups identifying poor countries, positive public expenditure effects are found but private expenditure effects are either non-significant or of comparable size to public effects. This outcome is partly sensitive to the methods used. The new dynamic panel model estimators introduced in this context, *LD*_*IV*_ and *KR*_*PRE*_ estimators, are not robust enough to provide an answer to the second question. We can observe like Kiviet et al. (2017, pp. 46–48) on *GMM* estimators that ‘However, not too much is known yet about the actual accuracy in practical situations on the abundance of different not always asymptotically equivalent implementations of estimators and test procedures”, and “Our results demonstrate that, especially under particular unfavorable settings, there is great urge for developing more refined inference procedures for structural dynamic panel data models’ to also be valid on *LD*_*IV*_ and *KR*_*PRE*_ estimators used here. For the *LD*_*IV*_, we need some guidance on how to determine the long difference length. *KR*_*PRE*_ needs some more robust developments under error heterogeneity and longer *AR* settings than *AR*(1).

The assumptions of variable strict exogeneity and predetermination made in the analysis are not harmless. Both the *LD*_*IV*_ and *KR*_*PRE*_ methods allow for settings where *HE* are endogenous (i.e. life expectancy and infant mortality determine the levels of private and public expenditures). This is not only the starting value or initial-level problem, but reasonable arguments can support the idea that the current levels of health status also determine *HE*. Typically, these arguments rest on the population’s age structure and on the ‘healthier are wealthier’ type of argument where a specific distinction is not made between private and public expenditures. On the contrary, our approach stressed the fact the *HE*_*PUB*_ are a policy-driven variable in the short run (i.e. some sort of exogeneity is a natural starting point in analysis). Some model estimations were conducted with endogenous *HE*, but they did not provide any results beyond what we have presented already. However, this important question must be addressed in detail in future research.

From a health policy perspective, the obtained results are interesting. In many country types analysed here there is still room for health improvements with larger *HE*. Globally, private expenditures seem less health-productive than *HE*_*PUB*_. Thus, the policy option would be a shift of resources from the private to the public sector. However, in practice this would be difficult by harming the consumer’s sovereignty with taxes and regulation. Also, first we must know in detail to what extent private and public health care services are complements or substitutes to each other. Note here that all three components of care—price, volume, and quality—have their impact on both the care provisions and their utilization. Typically, at least in non-developed countries, the price and quality components dominate in the private sector, as much of care provision here is based on imported medical skills and goods. As in many developed countries, care differentiation refers to the sector financing but not to the provision of health care, and the distinction between the two is not evident. In our data, high-income countries’ private and public expenditures correlate positively with each other much more strongly than in low-income countries (0.707 and 0.277, respectively). As the expenditure leakage is smaller in poor countries, correctly targeted *HE*_*PUB*_ and their marginal increases matter greatly in low-income countries. Thus, a general policy option is to subsidise the use of health care services so that the poorest can have access to and resources for them.
